# Improvement in Quality of Life With the Use of a Technological System Among Patients With Chronic Disease Followed Up in Primary Care (TeNDER Project): Protocol for a Randomized Controlled Trial

**DOI:** 10.2196/47331

**Published:** 2023-07-03

**Authors:** Cristina María Lozano Hernández, Rodrigo Medina-García, Mª Canto de Hoyos-Alonso, Araceli Garrido-Barral, César Minué Lorenzo, Teresa Sanz-Cuesta, Javier Serrano, Alberto del Rio Ponce, Tomas Gómez-Gascón, Isabel del Cura-González, TeNDER Group Primary Care

**Affiliations:** 1 Research Unit Primary Health Care Management Madrid Spain; 2 Biosanitary Research and Innovation Foundation of Primary Care Madrid Spain; 3 Research Network on Chronicity, Primary Care and Health Promotion Madrid Spain; 4 Primary Health Care Management General Ricardos Primary Health Care Centre Madrid Spain; 5 Primary Health Care Management Lain Entralgo Primary Health Care Centre Madrid Spain; 6 Primary Health Care Management Barrio del Pilar Primary Health Care Centre Madrid Spain; 7 Primary Health Care Management Perales del Río Primary Health Care Centre Madrid Spain; 8 Visual Telecommunications Application Research Group Signals, Systems and Radiocommunications Department Universidad Politécnica Madrid Spain; 9 Department of Medical Specialties and Public Health Faculty of Health Sciences Rey Juan Carlos University Madrid Spain; 10 See Acknowledgments

**Keywords:** chronic disease, environmental monitoring, technological development, quality of life, caregivers, health professionals, primary care, public health, monitoring, cognitive impairment, TeNDER

## Abstract

**Background:**

Among chronic diseases, cognitive, neurological, and cardiovascular impairments are becoming increasingly prevalent, generating a shift in health and social needs. Technology can create an ecosystem of care integrated with microtools based on biosensors for motion, location, voice, and expression detection that can help people with chronic diseases. A technological system capable of identifying symptoms, signs, or behavioral patterns could provide notification of the development of complications of disease. This would help the self-care of patients with chronic disease and save health care costs, promoting the autonomy and empowerment of patients and their caregivers, improving their quality of life (QoL), and providing health professionals with monitoring tools.

**Objective:**

The main objective of this study is to evaluate the effectiveness of a technological system (the TeNDER system) to improve quality of life in patients with chronic diseases: Alzheimer disease, Parkinson disease, and cardiovascular disease.

**Methods:**

A multicenter, randomized, parallel-group clinical trial will be conducted with a follow-up of 2 months. The scope of the study will be the primary care health centers of the Community of Madrid belonging to the Spanish public health system. The study population will be patients diagnosed with Parkinson disease, Alzheimer disease, and cardiovascular disease; their caregivers; and health professionals. The sample size will be 534 patients (380 in the intervention group). The intervention will consist of the use of the TeNDER system. The system will monitor the patients by means of biosensors, and their data will be integrated into the TeNDER app. With the information provided, the TeNDER system will generate health reports that can be consulted by patients, caregivers, and health professionals. Sociodemographic variables and technological affinity will be measured, as will views on the usability of and satisfaction with the TeNDER system. The dependent variable will be the mean difference in QoL score between the intervention and control groups at 2 months. To study the effectiveness of the TeNDER system in improving QoL in patients, an explanatory linear regression model will be constructed. All analyses will be performed with the 95% CI and robust estimators.

**Results:**

Ethics approval for this project was received on September 11, 2019. The trial was registered on August 14, 2020. Recruitment commenced in April 2021, and the expected results will be available during 2023 or 2024.

**Conclusions:**

This clinical trial among patients with highly prevalent chronic illnesses and the people most involved in their care will provide a more realistic view of the situation experienced by people with long-term illness and their support networks. The TeNDER system is in continuous development based on a study of the needs of the target population and on feedback during its use from the users: patients, caregivers, and primary care health professionals.

**Trial Registration:**

ClinicalTrials.gov NCT05681065; https://clinicaltrials.gov/ct2/show/NCT05681065

**International Registered Report Identifier (IRRID):**

DERR1-10.2196/47331

## Introduction

### Background

With the increase in population aging in Europe, chronic diseases are becoming more prevalent [1]. This reality represents a major challenge to health care systems due to comorbidities and outcomes such as increased dependency [2-4]. It is a priority to promote the autonomy and empowerment of patients and their caregivers, improve their quality of life (QoL), and provide tools enabling better monitoring by health professionals [5-7].

Among chronic diseases, cognitive, neurological, and cardiovascular impairments are becoming increasingly prevalent, generating a shift in health and social needs [8]. According to the World Health Organization (WHO) in a 2018 report, cognitive impairment and dementias, including Alzheimer disease (AD), remain one of the greatest global public health challenges facing our society [9]. The number of people worldwide living with a cognitive impairment such as dementia, is estimated at 44 million; this is expected to double by 2030 and is likely to increase to around 152 million by 2050. Among neurological disorders, Parkinson disease (PD) affects 1.2 million people in Europe [10]. Patients with PD, in addition to symptoms related to movement disorders, also develop cognitive impairment and emotional and behavioral disturbances that are important to follow closely [10]. Cardiovascular disease (CVD) accounted for 31% of all global deaths in 2016, and it is considered the leading cause of premature death (37% of all deaths under the age of 70) and disability in Europe and worldwide [11]. Early detection of disease progression and early treatment is essential [12].

Technology can create an ecosystem of care integrated with microtools based on biosensors for motion, location, voice, and expression detection that can help people with chronic diseases [13]. Today, these data streams, including sensor measurements, activity logs, and user-generated content, are used to measure or provide robust proxies for human behavior and function in both health and disease. This is called digital phenotyping [14].

Digital phenotyping is the in situ, moment-to-moment quantification of the human phenotype at the individual level using data from personal digital devices [15]. These microtools are able to recognize changes in a person’s habitual behaviors, movements, and mood through a multisensory system composed of smartphones, wearables, and other connected devices [16]. In addition, digital phenotyping allows the integration of an interactive communication service between health and social services, which can bring advantages in patient care and help detect risk situations, improving the autonomy of patients and the care they receive from caregivers and health professionals [17].

The development of a technological system capable of identifying symptoms, signs, or behavioral patterns that could provide alerts for the development of complications of these diseases would help the self-care of people with chronic diseases, their caregivers, and health professionals, as well as save costs for the health care system [18,19]. Some studies on digital phenotyping in patients with chronic disease have demonstrated its usefulness in treatment for mental health problems, diabetes, asthma, and CVD, showing positive effects on self-care and adherence to treatment [20,21]. This has been achieved by detecting clinical phenomena through digital tools that have enabled health professionals to adapt their assistance to the environment and characteristics of patients [21].

Until now, research on digital phenotyping has been led and developed by teams specialized in the technological field. As suggested by Huckvale et al [20], these challenges must be addressed under a multifaceted and multidisciplinary perspective involving health professionals and users in the development of such technological systems.

Needs such as the detection of behavioral changes in patients with dementia, gait changes and fall prevention in patients with PD, and the close monitoring of risk factors for CVD could be covered by intelligent monitoring system [19]. This would help the self-care of patients with chronic diseases, promoting the autonomy and empowerment of patients and their caregivers and improving their QoL.

### Objectives

The main objective of this study is to evaluate the effectiveness of a technological system (the TeNDER system) to improve QoL in patients with chronic diseases, including AD, PD, and CVD.

The secondary objectives are (1) to describe the satisfaction of patients, caregivers, and health professionals with the TeNDER system; (2) to describe the characteristics of the usability of the TeNDER system from the perspective of its potential users; and (3) to explore whether the use of the TeNDER system increases caregivers’ perceived satisfaction with the care provided and, in relation to this, improves their QoL.

## Methods

### Design

A multicenter, randomized, parallel-group clinical trial will be conducted with a follow-up of 2 months. The unit of randomization will be the patient.

This study protocol is part of a proposal with European funding under the European Union’s Horizon 2020 Research and Innovation Programme (grant 875325). The European consortium that developed the proposal is composed of different countries: Spain, Portugal, Italy, Slovenia, Greece, Germany, and Belgium [22].

The TeNDER project is developed in 4 European countries (Spain, Germany, Italy, and Slovenia). To account for the differences in the health systems of the participating countries and organizations, this research protocol refers to the primary care health centers (PCHCs) of the Madrid Health Service, which belongs to the Spanish public health system.

The TeNDER project brings together experts in technological development and users, including chronic patients, their caregivers, and primary care health professionals (PCHPs). Therefore, it will have an initial phase of cocreation in which the needs of the users will be explored. Based on the results, the technical team will work on improving and adapting the TeNDER system.

### Study Population

The TeNDER project will include patients diagnosed with at least one of the following chronic conditions: PD, AD, other dementias, and CVD. Along with the patients, their caregivers, and their health professionals from the PCHCs will also be included as participants.

All participants must accept and sign the informed consent form. If a patient does not have the capacity to do so, a family member will sign the form on their behalf.

### General Inclusion Criteria for Patients

Patients will be included if they are older than 60 years at follow-up, are referred by a PCHP at the PCHC, and are able to understand the local language. If a patient is dependent on others, they must have a caregiver or other responsible party.

### General Exclusion Criteria for Patients

Patient who according to the judgment of their caregiver or PCHP are unable to follow the requirements of the study (eg, patients with inability to move or move around at home) or who have a life expectancy of less than 6 months will be excluded.

### Specific Criteria for Patients With Each Chronic Illness

Patients with PD must have a confirmed diagnosis. Patients with AD must have a Mini Mental State Examination score between 19 and 28 points. Patients with a Global Deterioration Scale over 6 or 7 will be excluded. Patients with CVD will include those with the following conditions: heart failure with NYHA (New York Heart Association) Scale grade II to III heart function, and stable coronary artery disease or artery coronary disease with or without ST-elevation, atrial fibrillation, pacemaker, and stroke. Patients with arterial coronary disease that occurred less than 4 weeks ago or with severe aortic stenosis will be excluded.

### Inclusion Criteria for Caregivers

Caregivers of the selected patients who are aware of the patient’s health and social situation and who provide direct care or support for daily activities will be included. In addition, caregivers must be able to use a smartphone.

### Exclusion Criteria for Caregivers

Caregivers will be excluded if they do not understand the local language.

### Inclusion Criteria for PCHPs

Medical doctors and nurses attending the adult population of the participating PCHC will be included. They must carry out their professional activity in a PCHC that participates in the study and have at least one year of professional experience. They should be able to provide continuity of care for the patient during the intervention.

No exclusion criteria were applied for the PCHPs.

### Sample Size

The European consortium that developed the proposal called for a sample size of 1766 patients (1031 in the control group and 735 in the intervention group) with the aim of using the TeNDER system to achieve an improvement in QoL, as measured by the Short Form–36 Health Survey (SF36) [23], of 6 points in the intervention group as compared to the control group.

According to previous studies, the SD for QoL score in the population with the chronic diseases under study (PD, AD, and CVD) ranges between 16 and 27 [23-25]. For the power calculation, we used the highest value of this SD. Therefore, the sample size will allow a power of 98.8% to detect ≥6 differences in the overall score of the SF36 questionnaire, if they exist. Calculations were performed with Epidat (version 4.2; Xunta de Galicia).

In the PCHC of the Madrid Health Service, as part of the European consortium, 534 patients will be included (380 in the intervention group and 254 in the control group). No differentiation by pathology will be established.

### Recruitment

Five PCHCs in Madrid will be recruited. A total of 187 PCHPs will be invited to participate in the study. Based on experiences in previous studies, we expect a participation rate of 45%, which means 84 participating professionals. If necessary, the number of participating PCHCs will be expanded to reach the required sample size. PCHPs will participate in the study on a voluntary basis after signing the informed consent form.

Each participating PCHP will recruit 8 patients with a quota for each disease (5 with CVD, 1 with PD, and 2 with AD) consecutively as they attend the office. After the recruitment of patients, their caregivers will be invited to participate. For independent patients, it will not be necessary to recruit a caregiver.

During the recruitment interview, the PCHP will provide all information about the study as a detailed verbal explanation accompanied by a patient information sheet, making sure that the patient understands it.

### Randomization

Once the participants have been selected, after the initial visit, the patients will be randomized by pathology. Patients included in the intervention group will be included together with their caregiver and PCHP, creating a participant unit (patient-caregiver-PCHP or patient-PCHP). There will be no masking in any phase of the study. Assignment will be performed centrally by the Research Support Unit of the Primary Care Management of Madrid.

Patients in the intervention group will be fitted with the TeNDER system device best suited to their needs and will be treated according to standard clinical practice. Patients in the control group will be treated according to standard clinical practice.

Subsequently, each PCHP will be informed of which patients have been assigned to each group; based on this, they will have a different role in each case (ie, using the TeNDER system with the intervention group and usual practice with the control group).

### Intervention

**Figure 1:**
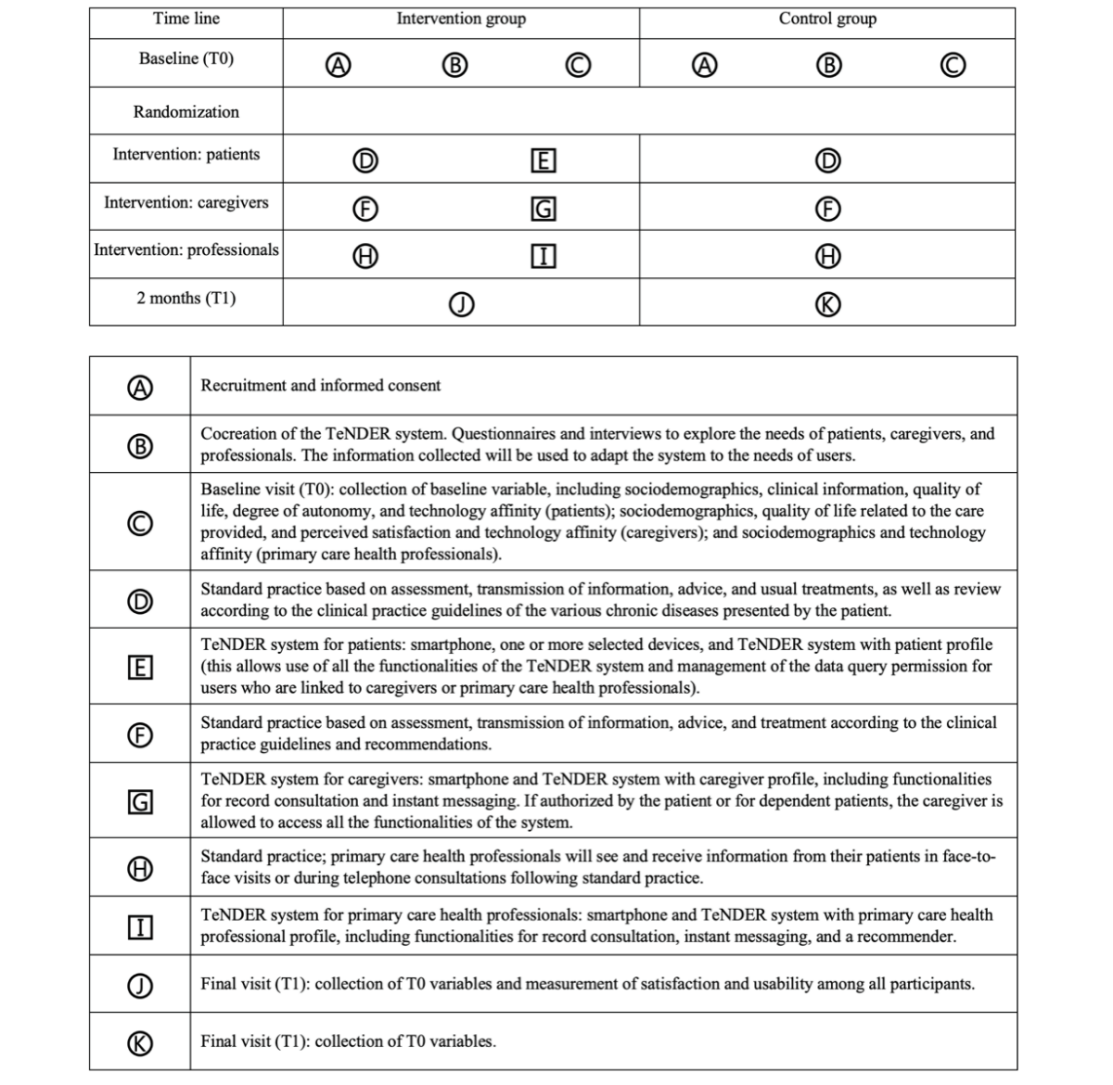
Details of patient flow, the intervention, and data collection.

The intervention is described in Figure 1 as recommended by Perera et al [26]. The intervention includes the use of the TeNDER system, a tool created by the consortium of the TeNDER group under the coordination of the Visual Telecommunications Applications Group (Grupo de Aplicación de Telecomunicaciones Visuales; GATV) of the Polytechnic University of Madrid.

The TeNDER system integrates multiple monitoring sensors that provide data to build an integrated technological ecosystem. The patient is monitored on a daily basis through one or more devices that offer different monitoring records (Table 1). The type of device will be chosen by the participant unit according to the patient’s health status and their needs as expressed by the patient and their caregiver.

With the registered information, the TeNDER system will generate different functionalities (Textbox 1) that can be used through a mobile app and a web app. The functionalities for each participant will be activated according to their profile. Patients will have access to all functionalities, while caregivers and professionals will only be able to access functionalities that the patient authorizes.

Each participant unit will receive assistance in downloading and installing the app, creating an account, and creating a user profile. The use of the TeNDER system does not require advanced knowledge, only a basic user level. However, technical support will be provided by the GATV of the Polytechnic University of Madrid.

**Table 1:** Devices and monitoring record.

Devices	Sleep quality detection	Emotional state detection	Physical activity^a^	Fall detection	Skeleton motion data^b^	Safety and security at home^c^
App for smartphones and tablets	✓	✓	✓	✓	✓	✓
Smartphone/tablet	✓	✓	✓	✓	✓	✓
Mini PC (low-end)	✓		✓	✓		
Mini PC (high-end)		✓			✓	✓
Speaker						
Smartband Fitbit			✓	✓	✓	✓
Environmental sensors (temperature and humidity)						✓
Microphone		✓				
Withings sleep analyzer (Withings France SA)	✓					
Localization sensor						✓
Open-close window and door sensors						✓
RGB camera (Intel RealSense)				✓	✓	

^a^Steps and heart rate.

^b^Balance, freezing, or both.

^c^Environmental status, door and window status, location, and activity at home.

**Functionalities of the TeNDER system.**

**Functionalities**

- Monitoring of variables collected by devices and biosensors
- Consultation of records and reports generated from the data collected by the biosensors
- Use of a smart calendar in which to schedule important events, medical appointment reminders, and medication reminders
- Possibility to generate notifications, including reminders and alerts
- Instant messaging channel for the exchange of messages between patient, caregiver, and primary care health professional
- Recommender function to explore a certain topic with the patient and, based on their answers, provide useful recommendations

### Data Collection

Two types of data will be collected based on the nature of the data: direct data (through questionnaires) and indirect data (through devices and biosensors).

#### Direct Data Collection

Once participants have agreed to participate in the study, they will be scheduled for the baseline visit (T0). At this visit, baseline and tool cocreation data will be collected. The final visit (T1) will take place 2 months after the intervention. Data collection during both visits will take place at the PCHC or at the patient’s home and will be carried out as face-to-face hetero-administered interviews by 2 project nurses. Data will be collected through questionnaires, and interviews will be indexed in an electronic data collection record (eDCR).

The primary outcome variable will be the QoL of patients measured using the SF36 questionnaire [23]. This is a 36-item questionnaire that includes 8 domains: physical functioning, role limitations due to physical problems, bodily pain, general health perceptions, mental health, role limitations due to emotional problems, social functioning, and vitality. Eight domain scores and a summary score (range 0-100) can be obtained; higher scores indicate better health-related QoL (HRQoL). In addition, six Likert-type questions are used to collect proxy indicators of QoL related to the TeNDER system. These questions are related to the perception of improved autonomy, support, improved self-care, and safety. In addition, we will collect sociodemographic variables such as sex, age, and nationality from patients, caregivers, and PCHPs. Also, technology affinity among the 3 types of users will be collected through a single ad hoc question. Clinical information collected from patients will include their main disease and other chronic diseases. The degree of autonomy and the need for help will be studied through ad hoc questionnaires at T0 and T1.

For a description of the usability of and satisfaction with TeNDER system after the intervention, the validated System Usability Scale questionnaire [24,25] will be administered to patients, caregivers, and PCHPs.

Caregivers’ QoL related to the care provided and the perceived satisfaction with the care provided will be studied through an ad hoc questionnaire.

The questionnaires (at T0 and T1) are included in Multimedia Appendix 1.

#### Indirect Data Collection

Indirect data collection will be performed through devices and biosensors. These data are transmitted to the TeNDER cloud platform using an intermediary broker messaging service (RabbitMQ). A mini PC, on which the local health tracking tool server is installed, serves as a gateway for the biosensors to communicate with the cloud.

To ensure seamless data collection, most sensors are connected to the local area network wirelessly. The biosensors collect a wide range of data, including the following:

- The Withings sleep analyzer captures sleep data, monitors heart rate and breathing rate, and is able to detect apnea states.
- The Smartwatch Fitbit Versa 2 detects heart rate and number of steps taken. In addition, raw accelerometer measurements extracted by the device help track fall incidents and patient location.
- A location sensor measures the signal strength of the smartwatch (via the Bluetooth connection) to determine position and presence at room level.
- A microphone and speaker provides real-time audio recording and is able to identify screams or cries through the collection of recordings and tone of voice.
- The Aqara Hub integrates several secondary devices, such as a room temperature and humidity sensor and binary door, window opening, and window closing sensors.
- The RealSense device captures the patient’s skeleton motion data in the form of coordinates in space. When combined with the Fitbit Versa 2 smartwatch, the system is able to identify the skeleton, and with the additional use of a microphone, can detect falls.

### Storage and Security of Collected Data

All the direct data will be stored in an eDCR on the EUSurvey platform. This platform is developed by the Commission of the European Union and complies with the European Union Regulation No 2018/1725 of the European Parliament and of the Council of 23 October 2018, by which it guarantees data privacy through its policy on the protection of natural persons with regard to the processing of personal data. Only project researchers will have access to the data stored in EUSurvey by means of a username and password. The data collected will be pseudoanonymized using an alphanumeric code, and only the researcher administering the questionnaire to the participant will be able to link these data to the participant. The data stored will remain in the database for up to 25 years after the end of data collection in accordance with current regulations.

As for indirect data, the TeNDER System platform is hosted on domains recommended and endorsed by the European Union, which guarantees the highest standards of security and privacy protection. Once collected, the data are temporarily stored on the device itself for a few days if there is no connection to the TeNDER system. After connection, the data are stored in an anonymized form in the secure infrastructure of the TeNDER system and can be accessed by project researchers using credentials and tokens.

### Data Analysis and Statistical Plan

A descriptive analysis of the characteristics of the participants will be performed with frequencies and percentages for qualitative variables and the mean (SD) or median (IQR) for the quantitative variables, according to their distribution. The mean difference in QoL score between the intervention and control groups will be studied with the corresponding 95% CIs at T1 (2 months). Differences in qualitative variables will be analyzed with the Pearson chi-squared test, and differences in normally distributed quantitative variables will be analyzed with the Student *t* test (2-tailed).

To study the effectiveness of the TeNDER System in improving QoL in patients, an explanatory linear regression model will be constructed. The dependent variable will be the mean difference in QoL score between the intervention and control groups at T1 (2 months). As patients will be recruited grouped by cluster (ie, PCHC), all the estimations will be performed with robust estimators. The analyses will be performed with Stata (version 14; StataCorp).

### Ethical Considerations

This study protocol was approved by the Ethics Committee for Research Involving Medicinal Products of the Hospital 12 de Octubre (20/450) and obtained permission from the Central Research Commission of the Primary Care Healthcare Management of Madrid (PC:39/20) to carry it out in the primary care setting of the Community of Madrid. The processing, communication, and transfer of data will be carried out in accordance with the provisions of the General Data Protection Regulation of the European Union (RGPD 2016/679) of the European Parliament and of the Council of 27 April 2016 and the Organic Law on Data Protection and Guarantee of Digital Rights in the Spanish territory (LOPDGDD 3/2018 of 5 December).

## Results

Ethics approval for this project was received on September 11, 2019. The trial was registered on August 14, 2020 (ClinicalTrials.gov NCT05681065). Recruitment commenced in April 2021 and the expected results will be available during 2023 or 2024.

## Discussion

Previous studies on the use of technological tools by people with chronic disease have shown an improvement in their self-management, self-care, and adherence to therapy. However, few studies have examined all users involved in the care of chronic patients in their use of these tools.

The strength of this work is to investigate the use of a technological tool focused on health and self-care by, on one hand, patients with chronic disease older than 65 years, and on the other hand, their caregivers or family supporters, as well as, finally, the health professionals who are responsible for monitoring their health status, in particular the PCHPs. This clinical trial among patients with highly prevalent chronic diseases and the people most involved in their care will provide a more realistic view of how technology can be integrated into their lives as an aid to care.

Another strong point is the involvement of users in the development and improvement of the TeNDER system. To this end, this work includes a cocreation phase in which the needs of the users are explored in order to develop a system that is adapted to them.

The main objective of this study is to improve the QoL of chronically ill patients older than 60 years. Despite the difficulties of achieving this with a 2-month intervention, there are certain proxy indicators of QoL that are sensitive to improvement, and this can be reflected in the HRQoL. Therefore, ad hoc questions have been included as variables that aim to detect elements that have an impact on QoL, such as safety and autonomy.

Among the main limitations are that due to the nature of the intervention, the trial cannot be masked. This may result in participants who are in the intervention group perceiving an improvement in their health follow-up; therefore, the difference in score with respect to the control group will increase. Thus, we propose to look for an increase of at least 6 points in the main improvement variable (QoL). In the same sense, the use of the TeNDER system can also lead to an improvement in patients’ adherence to treatment, as they feel observed and monitored by the system (ie, the Hawthorne effect).

Another limitation is selection bias in the sample of participants. In many cases, older people and people with low digital literacy have the greatest need for health support. However, since the intervention requires the use of technology, it may be those with the greatest interest in its use who agree to participate in the study. This may result in a missed opportunity to address the digital divide. Studying the effectiveness of a technological system in an older population (ie, patients aged >60 years) may imply an initial reluctance toward technology. The digital age gap can be a barrier to use, and as a consequence, this can affect satisfaction and usability.

In order to reduce this effect and to be able to achieve good satisfaction and adapt the system to the participating population, the TeNDER system is developed based on a study of the needs of the target population (patients, caregivers, and PCHPs) and is constantly improved based on feedback from them.

In addition, in order to reduce the frustration that may arise with the use of technology, continuous telephone support will be provided to address any doubts or problems that may arise for participants.

## Appendix

Multimedia Appendix 1 Questionnaires administered at T0 and T1 for patients, caregivers, and primary care health professionals.

## Abbreviations

AD: Alzheimer disease
CVD: cardiovascular disease
eDCR: electronic data collection record
EU: European Union
GATV: Grupo de Aplicación de Telecomunicaciones Visuales
HRQoL: health-related QoL
NYHA: New York Heart Association
PCHC: primary care health center
PCHP: primary care health professional
PD: Parkinson disease
QoL: quality of life
SF36: Short Form–36
WHO: World Health Organization

## Data Availability

Data sharing is not applicable to this paper as no data sets were generated or analyzed during this study.
