# Supplementary material for: Improvement in Quality of Life With the Use of a Technological System Among Patients With Chronic Disease Followed Up in Primary Care (TeNDER Project): Protocol for a Randomized Controlled Trial
Source: JMIR Res Protoc. 2023 Jul 3;12:e47331. doi: 10.2196/47331 (PMC10365573; doi:10.2196/47331)
Supplement: Multimedia Appendix 1 [file resprot_v12i1e47331_app1.docx]

## Annex 1. Questionnaires administered in T0 and T1 for patients, caregivers, and primary care health professionals.

### Short Form-36 Health Survey (SF36)

| **GENERAL HEALTH** | | | | | | | | | | | | | | | | | | | | | | | |
| --- | --- | --- | --- | --- | --- | --- | --- | --- | --- | --- | --- | --- | --- | --- | --- | --- | --- | --- | --- | --- | --- | --- | --- |
| 1. In general, would you say your health is: | | | | | | | | | | | | | | | | | | | | | | | |
|  | Excellent | | | Very good | | | | | Good | | | Fair | | | | | | Poor | | | | | |
|  |  | | |  | | | | |  | | |  | | | | | |  | | | | | |
| 1. Compared to one year ago, how would you rate your health in general now? | | | | | | | | | | | | | | | | | | | | | | | |
|  | Much better now than one year ago | | | Somewhat better now than one year ago | | | | | About the same | | | Somewhat worse now than one year ago | | | | | | Much worse than one year ago | | | | | |
|  |  | | |  | | | | |  | | |  | | | | | |  | | | | |  |
| **LIMITATIONS OF ACTIVITIES** | | | | | | | | | | | | | | | | | | | | | | | |
| The following items are about activities you might do during a typical day. Does your health now limit you in these activities. If so, how much? | | | | | | | | | | | | | | | | | | | | | | | |
|  |  | | |  | | | |  | Yes, limited a lot | | | | | Yes, limited a little | | | | | | No, not limited at all | | | |
| 1. Vigorous activities, such as running, lifting heavy objects, participating in strenuous sports. | | | | | | | | |  | | | | |  | | | | | |  | | | |
| 1. Moderate activities, such as moving a table, pushing a vacuum cleaner, bowling, or playing golf | | | | | | | | |  | | | | |  | | | | | |  | | | |
| 1. Lifting or carrying groceries | | | | | | | | |  | | | | |  | | | | | |  | | | |
| 1. Climbing several flights of stairs | | | | | | | | |  | | | | |  | | | | | |  | | | |
| 1. Climbing one flight of stairs | | | | | | | | |  | | | | |  | | | | | |  | | | |
| 1. Bending, kneeling, or stooping | | | | | | | | |  | | | | |  | | | | | |  | | | |
| 1. Walking more than a mile | | | | | | | | |  | | | | |  | | | | | |  | | | |
| 1. Walking several blocks | | | | | | | | |  | | | | |  | | | | | |  | | | |
| 1. Walking one block | | | | | | | | |  | | | | |  | | | | | |  | | | |
| 1. Bathing or dressing yourself | | | | | | | | |  | | | | |  | | | | | |  | | | |
| **PHYSICAL HEALTH PROBLEMS** | | | | | | | | | | | | | | | | | | | | | | | |
| During the past 4weeks, have you had any of the following problems with your work or other regular daily activities as a result of your physical health? | | | | | | | | | | | | | | | | | | | | | | | |
|  |  | | |  | | | | |  | | |  | | | | | | Yes | | | | No | |
| 1. Cut down the amount of time you spent on work or other activities | | | | | | | | | | | | | | | | | |  | | | |  | |
| 1. Accomplished less than you would like | | | | | | | | | | | | | | | | | |  | | | |  | |
| 1. Were limited in the kind of work or other activities | | | | | | | | | | | | | | | | | |  | | | |  | |
| 1. Had difficulty performing the work or other activities (for example, it took extra effort) | | | | | | | | | | | | | | | | | |  | | | |  | |
| **EMOTIONAL HEALTH PROBLEMS** | | | | | | | | | | | | | | | | | | | | | | | |
| During the past 4 weeks, have you had any of the following problems with your work or other regular daily activities as a result of any emotional problems (such as feeling depressed or anxious)? | | | | | | | | | | | | | | | | | | | | | | | |
|  |  | | |  | | | | |  | | |  | | | | | | Yes | | | | No | |
| 1. Cut down the amount of time you spent on work or other activities | | | | | | | | | | | | | | | | | |  | | | |  | |
| 1. Accomplished less than you would like | | | | | | | | | | | | | | | | | |  | | | |  | |
| 1. Didn’t do work or other activities as carefully as usual | | | | | | | | | | | | | | | | | |  | | | |  | |
| **SOCIAL ACTIVITIES** | | | | | | | | | | | | | | | | | | | | | | | |
| 1. Emotional problems interfered with your normal social activities with family, friends, neighbors, or groups? | | | | | | | | | | | | | | | | | | | | | | | |
|  | | Not at all | | Slightly | | | Moderately | | | | | | Severe | | | | | Very severe | | | | | |
|  | |  | |  | | |  | | | | | |  | | | | |  | | | | |  |
| **PAIN** | | | | | | | | | | | | | | | | | | | | | | | |
| 1. How much bodily pain have you had during the past 4 weeks? | | | | | | | | | | | | | | | | | | | | | | | |
|  | | None | | Very mild | | | Mild | | | | Moderate | | | | | Severe | | | | | Very severe | | |
|  | |  | |  | | |  | | | |  | | | | |  | | | | |  | | |
| 1. During the past 4 weeks, how much did pain interfere with your normal work (including both work outside the home and housework)? | | | | | | | | | | | | | | | | | | | | | | | |
|  | | | Not at all | A little bit | | | Moderately | | | | | Quite a bit | | | | | | | Extremely | | | | |
|  | | |  |  | | |  | | | | |  | | | | | | |  | | | |  |
|  | | | | | | | | | | | | | | | | | | | | | | | |
| **ENERGY AND EMOTIONS** | | | | | | | | | | | | | | | | | | | | | | | |
| These questions are about how you feel and how things have been with you during the last 4 weeks. For each question, please give the answer that come closest to the way you have been feeling. | | | | | | | | | | | | | | | | | | | | | | | |
|  | | | | Al lot of the time | | Most of the time | | | A good bit of the time | | | Some of the time | | | | | A little bit of the time | | | | None of the Time | | |
| 1. Did you feel full of pep? | | | |  | |  | | |  | | |  | | | | |  | | | |  | | |
| 1. Have you been a very nervous person? | | | |  | |  | | |  | | |  | | | | |  | | | |  | | |
| 1. Have you felt so down in the dumps that nothing could cheer you up? | | | |  | |  | | |  | | |  | | | | |  | | | |  | | |
| 1. Have you felt calm and peaceful? | | | |  | |  | | |  | | |  | | | | |  | | | |  | | |
| 1. Did you have a lot of energy? | | | |  | |  | | |  | | |  | | | | |  | | | |  | | |
| 1. Have you felt downhearted and blue? | | | |  | |  | | |  | | |  | | | | |  | | | |  | | |
| 1. Did you feel worn out? | | | |  | |  | | |  | | |  | | | | |  | | | |  | | |
| 1. Have you been a happy person? | | | |  | |  | | |  | | |  | | | | |  | | | |  | | |
| 1. Did you feel tired? | | | |  | |  | | |  | | |  | | | | |  | | | |  | | |
| **SOCIAL ACTIVITIES** | | | | | | | | | | | | | | | | | | | | | | | |
| 1. During the past 4 weeks, how much of the time has your physical health or emotional problems interfered with your social activities (like visiting with friends, relatives, etc.)? | | | | | | | | | | | | | | | | | | | | | | | |
|  | Al lot of the time | | | Most of the time | | | | | Some of the time | | | | | A Little bit of the time | | | | | | Never | | | |
|  |  | | |  | | | | |  | | | | |  | | | | | |  | | |  |
| **GENERAL HEALTH** | | | | | | | | | | | | | | | | | | | | | | | |
| How true or false is each of the following statements for you? | | | | | | | | | | | | | | | | | | | | | | | |
|  | | | | | Definitely true | | Mostly true | | | Don’t know | | | | | Mostly false | | | | Definitely false | | | | |
| 1. I seem to get sick a little easier than other people | | | | |  | |  | | |  | | | | |  | | | |  | | | |  |
| 1. I am as healthy as anybody I know | | | | |  | |  | | |  | | | | |  | | | |  | | | |  |
| 1. I expect my health to get worse | | | | |  | |  | | |  | | | | |  | | | |  | | | |  |
| 1. My health is excellent | | | | |  | |  | | |  | | | | |  | | | |  | | | |  |

SF36 Questionnaire provide by RAND Corporation <https://https://www.rand.org/health-care/surveys_tools/mos/36-item-short-form/survey-instrument.html>

The RAND Corporation is a research organization that develops solutions to public policy challenges to help make communities throughout the world safer and more secure, healthier, and more prosperous. RAND is nonprofit, nonpartisan, and committed to the public interest. RAND is a registered trademark.

### Technology affinity ad hoc questionnaire.

| 1. I like testing the functions of new technical systems | | | | | | |
| --- | --- | --- | --- | --- | --- | --- |
|  | Completely agree | Largely agree | Slightly agree | Slightly agree | Largely agree | Completely agree |
|  |  |  |  |  |  |  |

### Degree of Autonomy and need for help ad hoc questionnaire.

| The following items are about activities you might do during a typical day. Does your health now limit you in these activities. If so, how much? | | | | | |
| --- | --- | --- | --- | --- | --- |
|  | Always | Often | Sometimes | Rarely | Never |
| 1. How often do you require help from other persons in your daily activities? |  |  |  |  |  |
| 1. How often do you postpone doing things as you don’t feel confident? |  |  |  |  |  |
| 1. How often do you confidently go out of your apartment/house? |  |  |  |  |  |
| 1. How often do you feel lost? |  |  |  |  |  |
| 1. How often do you call services to help you? |  |  |  |  |  |
| 1. How often do you feel safe at home? |  |  |  |  |  |

### System Usability Scale (SUS) questionnaire

|  |  |  | Strongly disagree |  |  |  | Strongly agree |
| --- | --- | --- | --- | --- | --- | --- | --- |
| 1. | I think that I would like to use this system frequently |  |  |  |  |  |  |
|  |  |  | 1 | 2 | 3 | 4 | 5 |
| 2. | I found the system unnecessarily complex |  |  |  |  |  |  |
|  |  |  | 1 | 2 | 3 | 4 | 5 |
| 3. | I thought the system was easy to use |  |  |  |  |  |  |
|  |  |  | 1 | 2 | 3 | 4 | 5 |
| 4. | I think I would need the support of a technical person to be able to use this system |  |  |  |  |  |  |
|  |  |  | 1 | 2 | 3 | 4 | 5 |
| 5. | I found the various functions in this system to be well integrated |  |  |  |  |  |  |
|  |  |  | 1 | 2 | 3 | 4 | 5 |
| 6. | I thought there was too much inconsistency in this system |  |  |  |  |  |  |
|  |  |  | 1 | 2 | 3 | 4 | 5 |
| 7. | I would imagine that most people would learn to use this system very quickly |  |  |  |  |  |  |
|  |  |  | 1 | 2 | 3 | 4 | 5 |
| 8. | I found the system very awkward to use |  |  |  |  |  |  |
|  |  |  | 1 | 2 | 3 | 4 | 5 |
| 9. | I felt very confident using the system |  |  |  |  |  |  |
|  |  |  | 1 | 2 | 3 | 4 | 5 |
| 10. | I needed to learn a lot of things before I could get going with this system |  |  |  |  |  |  |
|  |  |  | 1 | 2 | 3 | 4 | 5 |

### Quality of Life related to the care provided ad hoc questionnaire.

| 1. How would you rate your quality of life? | | | | | |
| --- | --- | --- | --- | --- | --- |
|  | Always | Often | Sometimes | Rarely | Never |
|  |  |  |  |  |  |
| 1. How often do you feel calm and relaxed? | | | | | |
|  | Always | Often | Sometimes | Rarely | Never |
|  |  |  |  |  |  |
| 1. How do you feel when you are not with the person who you are caring for? | | | | | |
|  | Very insecure | Insecure | A Little insecure | Safe | Very safe |
|  |  |  |  |  |  |
| 1. How often do you need to check for his/her needs in a day? | | | | | |
|  | Always | Often | Sometimes | Rarely | Never |
|  |  |  |  |  |  |
| 1. How often do you need to visit/to attend the person who you are caring for? | | | | | |
|  | Always | Often | Sometimes | Rarely | Never |
|  |  |  |  |  |  |
| 1. How would you rate your physical status? | | | | | |
|  | Very poor | Poor | Normal | Good | Excellent |
|  |  |  |  |  |  |
| 1. Do you feel the person you are caring for is dependent on you? | | | | | |
|  | Always | Often | Sometimes | Rarely | Never |
|  |  |  |  |  |  |
| 1. Do you feel stressed between caring for the person you take care for and trying to meet other responsibilities for your family or work? | | | | | |
|  | Always | Often | Sometimes | Rarely | Never |
|  |  |  |  |  |  |
| 1. Do you feel your health has suffered because of your involvement with the person you are caring for? | | | | | |
|  | Always | Often | Sometimes | Rarely | Never |
|  |  |  |  |  |  |
| 1. Do you feel you could do better in taking care of the person you care? | | | | | |
|  | Always | Often | Sometimes | Rarely | Never |
|  |  |  |  |  |  |
| 1. How often do you feel your cared person is safe at home when you leave it? | | | | | |
|  | Always | Often | Sometimes | Rarely | Never |
|  |  |  |  |  |  |

### Perceived satisfaction with the care provided ad hoc questionnaire.

| 1. How do you feel about his/her care? | | | | | |
| --- | --- | --- | --- | --- | --- |
|  | Very satisfied | Satisfied | Neutral | Unsatisfied | Very unsatisfied |
|  |  |  |  |  |  |

### Time saving using TeNDER system.

|  | Yes | I don’t know | No |
| --- | --- | --- | --- |
| 1. Do you think that TeNDER system could help you to reduce the number of unnecessary visits? |  |  |  |
| 1. Do you think that TeNDER system leads to a potential decrease of time in access to patient clerical information? |  |  |  |

### Usefulness perceived after using TeNDER system.

|  | Yes | I don’t know | No |
| --- | --- | --- | --- |
| 1. Do you get more information about him/her and find this useful? |  |  |  |
| 1. Does TeNDER system improve your approach to the patient? |  |  |  |
| 1. Have you found something new that improves your knowledge of him/her using TeNDER system? |  |  |  |
| 1. Do you feel that you can apply better or more specific therapies to the patient because of the system? |  |  |  |
